# Invasion of Dendritic Cells, Macrophages and Neutrophils by the *Bordetella* Adenylate Cyclase Toxin: A Subversive Move to Fool Host Immunity

**DOI:** 10.3390/toxins9100293

**Published:** 2017-09-21

**Authors:** Giorgio Fedele, Ilaria Schiavoni, Irena Adkins, Nela Klimova, Peter Sebo

**Affiliations:** 1 Department of Infectious Diseases, Istituto Superiore di Sanità, Rome 00161, Italy; ilaria.schiavoni@iss.it; 2 Sotio a.s, Prague170 00, Czech Republic; adkins@sotio.com; 3 Department of Immunology, 2nd Faculty of Medicine, Charles University and University Hospital Motol, Prague 150 00, Czech Republic; 4 Institute of Microbiology of the CAS, v. v. i., Prague 142 20, Czech Republic; nelaklim@biomed.cas.cz (N.K.); sebo@biomed.cas.cz (P.S.); 5 Faculty of Science, Charles University in Prague, Prague 128 00, Czech Republic

**Keywords:** immune response, intracellular pathways, phagocytosis, T-helper cells

## Abstract

Adenylate cyclase toxin (CyaA) is released in the course of *B. pertussis* infection in the host’s respiratory tract in order to suppress its early innate and subsequent adaptive immune defense. CD11b-expressing dendritic cells (DC), macrophages and neutrophils are professional phagocytes and key players of the innate immune system that provide a first line of defense against invading pathogens. Recent findings revealed the capacity of *B. pertussis* CyaA to intoxicate DC with high concentrations of 3′,5′-cyclic adenosine monophosphate (cAMP), which ultimately skews the host immune response towards the expansion of Th17 cells and regulatory T cells. CyaA-induced cAMP signaling swiftly incapacitates opsonophagocytosis, oxidative burst and NO-mediated killing of bacteria by neutrophils and macrophages. The subversion of host immune responses by CyaA after delivery into DC, macrophages and neutrophils is the subject of this review.

## 1. Introduction

The whooping cough agent *Bordetella pertussis* is endowed with an arsenal of virulence factors. This includes several protein toxins (adenylate cyclase toxin, pertussis toxin, dermonecrotic toxin and the Type III secreted effector BteA/BopC) and numerous adhesins and autotransporter surface proteins (e.g., fimbriae, FHA, Tcf, pertactin), involved in infection and colonization of the host [1]. The adenylate cyclase toxin-hemolysin (ACT, AC-Hly or CyaA) is a key virulence factor of *B. pertussis*, specifically regulated by the *Bordetella* virulence gene system. CyaA plays a particular role in the early phases of airway colonization and its capacity to instantly ablate the bactericidal oxidative burst and opsonophagocytic killing capacities of neutrophils and macrophages enables establishment of *Bordetella* infection of airway mucosa [2,3,4,5,6]. The CyaA toxin was first described in 1976 by Hewlett in *B. pertussis* cultures as a soluble adenylyl cyclase (AC) enzyme (EC 4.6.1.1) converting ATP to cAMP (3′,5′-cyclic adenosine monophosphate), a key intracellular ‘second messenger’ molecule of eukaryotic cells [7]. Subsequently, the AC enzyme was also detected in several commercial whole cell pertussis vaccine preparations [8]. Since then, hundreds of articles elaborated on the expression, structure, mode of action, role in virulence and use in antigen delivery into DCs of this unique multifunctional RTX family toxin. For mechanistic aspects of CyaA action and its potential for use in pertussis vaccines, the reader is referred to a dedicated recent review [9].

## 2. Immunity to *B. pertussis*

Colonization of the mucosal surface of the host respiratory tract by *B. pertussis* triggers an immune response, in which multiple bacterial molecules engage the pathogen recognition receptors expressed by both epithelial cells and resident antigen-presenting cells. Pathogen recognition activates the primary innate immune defense and shapes the initial local adaptive immune response to *B. pertussis*. At this stage, the immune effector cells release cytokines with local pro- or anti-inflammatory effects. As the infection progresses, bacteria are challenged by the adaptive immune response that is primarily activated by the dendritic cells (DC) and by resident alveolar macrophages. In particular, immature intraepithelial DC reside in the mucosa, where they act as sentinels of the immune system. After recognition of bacterial toll-like receptor (TLR) ligands, these DC undergo a profound rearrangement of gene expression that leads to a different cell subtype, the mature DC. This acquires the ability to migrate to secondary lymphoid tissue, the lymph nodes and follicles of the mucosa-associated lymphoid tissue and to the spleen, where the DC presents pathogen-derived antigens to naïve CD4^+^ and CD8^+^ T cells to initiate the adaptive T cell immune response. 

The immunological synapse with the antigen-presenting DC primes the differentiation and expansion of naïve T cells, which may differentiate into different subsets of T-helper (Th) cells. Typically, Th1 cells produce interferon (IFN)-γ, tumor necrosis factor (TNF)-α and interleukin (IL)-2 and play an important role in cell-mediated inflammatory responses [10]. The Th2 cells are then characterized by the secretion of high levels of IL-4, IL-5, and IL-13 and are critical for the development of antibody responses [11]. Th17 cells, instead, expressing IL-17, IL-21, and IL-22, have been implicated in host inflammatory responses against various bacterial pathogens and appear to play a crucial role in defense against the pathogens in airway mucosa [12,13]. The local cytokine environment during priming, together with the antigen dose, antigen affinity, major histocompatibility complex haplotypes and the costimulatory molecules expressed by the mature DC [14], determines the fate of T cell differentiation. An important subset of CD4^+^ T cells licensed by mature DC in the secondary lymph nodes is represented by regulatory T cells (Tregs). Unlike other T cell subsets that promote an immune response, Tregs are immunosuppressive and have the function of maintaining self-tolerance. The suppressive activity of Tregs mitigates immune responses against infectious agents and is pivotal in re-establishing immune homeostasis following pathogen clearance [15]. A wide array of genes are typically expressed by Tregs including cytokines, such as IL-10 and transforming growth factor (TGF)-β, and surface molecules, such as cytotoxic T lymphocyte-associated antigen 4 (CTLA-4), glucocorticoid-induced TNF receptor family-related gene, CD27 and lymphocyte activation gene-3 (LAG-3). Moreover, Tregs classically express Foxp3, a transcription factor recognized as the master regulator essential for their functions [16]. Recent studies led to the identification of new Th cell subsets, namely Th9, Th22 and Thf cells. Th9 cells play an important role in allergy and autoimmunity, Th22 in skin inflammation and repair and Thf in B cell priming and maintenance of humoral memory [17,18,19]. The cytokines produced by mature DC during T cell activation have a strong influence on the differentiation of Th cells subsets. High levels of IL-12, for example, promote the expansion of Th1 cells, while simultaneously inhibiting Th2 cell proliferation. Conversely, high levels of IL-4 induce Th2 cells and suppress Th1 expansion [20,21]. Typical mediators for the expansion of the Th17 subtype are IL-1β, IL-6, and IL-23 [22,23]. Recent studies have shown that CD4^+^ Th cell subsets display a certain degree of plasticity. For instance, it is known that Th17 cells can undergo an IL-12-dependent transition to the Th1 state [24] and that Tregs can evolve into Th17 cells in the presence of IL-6 and TGF-β [25].

Cellular immunity against *B. pertussis* has been attributed to CD4^+^ T lymphocytes [26,27], although the role of CD8^+^ T cells remains unexplored and cannot be excluded [28]. Studies in the murine respiratory tract infection model demonstrated a dominant role for IFN-γ secreting Th1 cells [26]. The requirement for a Th1 polarized response to achieve protection has also been shown in population studies in humans [29,30,31]. More recently, the ability of *B. pertussis* to skew the host immune response towards the expansion of Th17 cells was observed. In mice, pertussis infection or immunization with the whole cell vaccine induce a Th17 response and the generation of antigen-specific Th17 cells correlates with protection [32,33,34,35]. In agreement with these results, it has been shown that human monocyte-derived DC infected ex vivo with *B. pertussis* induce a mixed Th1/Th17 polarization of CD4^+^ T cells [36,37]. Collectively, these data suggest that *Bordetella* infection may induce a mixed Th1/Th17-polarized immune response in the host. 

Many pathogens have developed the ability to interfere with host immune response to escape clearance. To this aim, bacteria express virulence factors that can manipulate the functions of host’s cellular machineries devoted to initiation of an appropriate immune response [38]. *B. pertussis* has evolved many different strategies of immune evasion, which include avoidance of a proper recognition by pathogen recognition receptors [39], complement resistance [40,41], manipulation of immune cells by pertussis toxin [42,43,44] increase of intracellular survival [45,46] or interference with the activation of inflammatory signals [47,48]. The secreted adenylate cyclase toxin (CyaA) of *B. pertussis* then plays a major role in subversion of the functions of immune cells and promotes immune evasion of *B. pertussis*. 

## 3. CyaA Mode of Action on Host Cells

**Figure 1:**
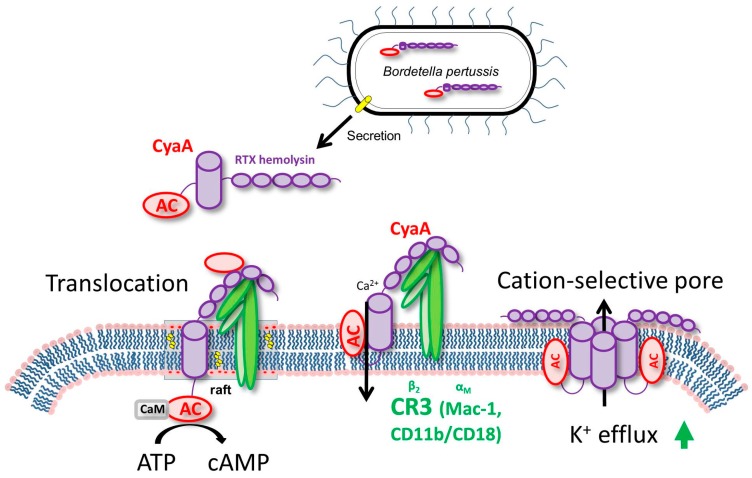
Mode of action of adenylate cyclase toxin (CyaA) on phagocyte membrane. CyaA is secreted from the bacterium into the calcium-containing serous fluid on mucosal surfaces through the Type I secretion system (T1SS). It binds calcium ions and co-secretionally folds into a conformation capable to bind the complement receptor 3 (CR3), known as the α_M_β_2_ integrin, CD11b/CD18 or Mac-1, on the surface of myeloid phagocytic cells [49,50,51]. Concentration and positioning of the toxin on the surface of the cell enables its insertion into the lipid bilayer of cellular membrane. The translocation precursor of CyaA generates a path for influx of extracellular calcium ions into cells [52], which leads to activation of calpain-mediated cleavage of talin [52] and mobilization of the CyaA–CR3 complex into lipid rafts [53]. From there the AC domain translocates across cellular membrane into the submembrane region of cells, where signaling complexes including protein kinase A are clustered [53,54]. The AC enzyme binds cytosolic calmodulin and catalyzes unregulated conversion of ATP into the key second messenger molecule 3′,5′-cyclic adenosine monophosphate (cAMP). In parallel, the RTX hemolysin part of CyaA is functionally independent of the invasive AC domain and the pore-forming precursors form oligomeric cation-selective pores that permeabilize cellular membrane for efflux of cytosolic potassium ions from cells [54]. See parallel review by Novak et al. in this issue for further mechanistic details on CyaA action (adapted from Masin et al. [55]).

CyaA, a 1706 residue long bi-functional leukotoxin, is endowed with a cell-invasive N-terminal adenylyl cyclase (AC) enzyme domain (~400 residues) that is fused to a pore-forming RTX (repeat in toxin) ‘cytolysin/hemolysin’ moiety of ~1300 residues. CyaA binds the complement receptor 3 (CR3), the α_M_β_2_ integrin known also as CD11b/CD18 or Mac-1 and the toxin translocates its AC enzyme domain across the cytoplasmic membrane of CR3-expressing myeloid cells, such as macrophages, neutrophils and dendritic cells [49,50,51] (Figure 1). 

CyaA insertion into the membrane of phagocytes induces an influx of calcium ions that leads to a calpain-mediated cleavage of talin [52,53,54]. This mobilizes the CyaA–CR3 complex for relocation into lipid rafts. The AC domain is translocated across the cellular membrane into the transmembrane region, where signaling complexes including protein kinase A are clustered [52,53,54]. Inside cells, the AC enzyme binds calmodulin and catalyzes unregulated conversion of ATP into the key second messenger molecule 3′,5′-cyclic adenosine monophosphate (cAMP), which incapacitates bactericidal activities of the target cells. In parallel, the RTX hemolysin part of CyaA is functionally independent of the invasive AC domain and forms oligomeric cation-selective pores that permeabilize cellular membranes for efflux of cytosolic potassium ions from cells [54] (Figure 1). 

## 4. CyaA Interference with Innate Immune Defense

**Figure 2:**
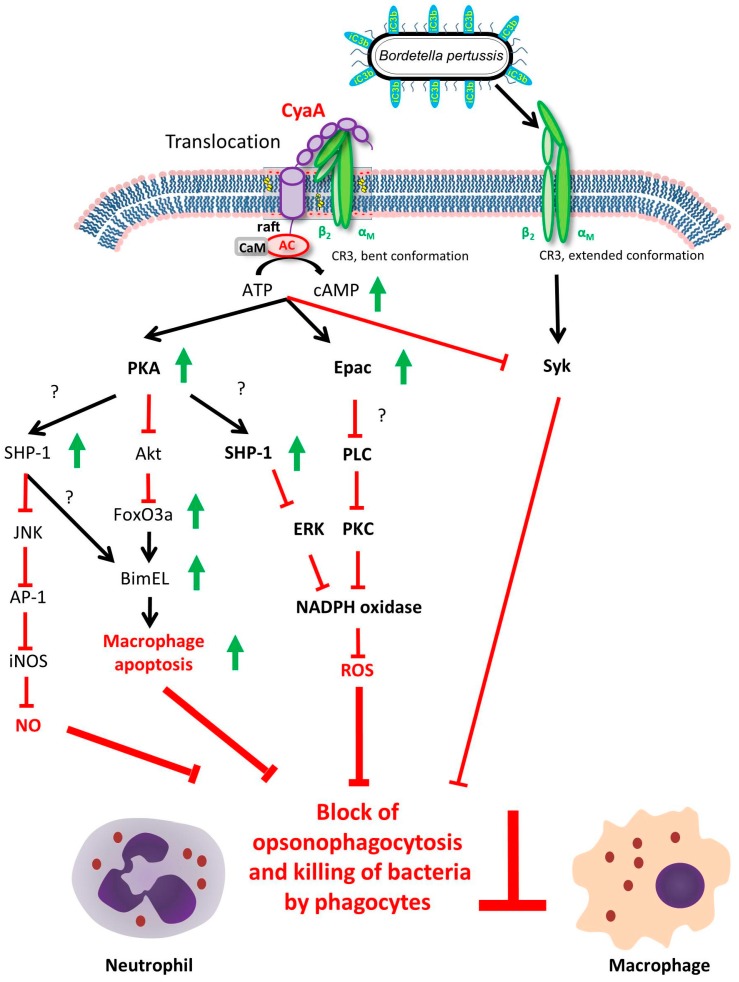
**Hijacking of cellular signaling cascades blocks the bactericidal activities of phagocytes.** Elevation of cAMP concentration in the cytosol of CR3-expressing myeloid phagocytes activates a wealth of signaling cascades that converge towards ablation of bactericidal activities of phagocytes, such as oxidative burst and phagocytic uptake and killing of opsonized bacteria. By an as-yet unknown mechanism, CyaA/cAMP signaling inhibits the activity of the Syk kinase and blocks, thereby, CR3 signaling triggered by binding of iC3b-opsonized bacteria to the open (extended) CR3 receptor [59]. In parallel, cAMP activates the protein kinase A (PKA) and the exchange protein directly activated by cAMP (Epac) signaling pathways, interfering with numerous physiological signaling processes (see parallel in this issue the in-depth review of CyaA-triggered signaling by Novak et al.). In particular, PKA mediates by an as yet undefined mechanism the activation of the Src homology domain 2 containing protein tyrosine phosphatase (SHP) 1 [63]. This provokes inhibition of ERK1/2 and together with Epac-mediated inhibition of phospholipase C (PLC) inhibits the assembly of the NADPH oxidase, production of reactive oxygen species (ROS) and oxidative burst of neutrophils and macrophages [6,61]. In macrophages, the activation of SHP-1 yields dephosphorylation of the c-Fos subunit of the transcription factor AP-1 and blocks expression of TLR-inducible NO synthase (iNOS), which enables the internalized non-opsonized *B. pertussis* bacteria to evade NO-mediated intracellular killing [45,63]. Lastly, activation of SHP-1, inhibition of the pro-survival kinase Akt/PKB and inhibition of ERK1/2 act together to abrogate degradation of BimEL. Enhanced BimEL levels then activate Bax and trigger apoptosis of macrophages through the mitochondrial pathway [64].

Phagocytosis by alveolar macrophages and by neutrophils arriving to the airway mucosa is the first step towards the elimination of pathogens from the respiratory tract. The near-instant production of very high levels of intracellular cAMP in CD11b-expressing immune cells that encounter *B. pertussis*-produced CyaA then rapidly interferes with the physiological functions of phagocytes [2,50]. This occurs primarily through activation of the protein kinase A (PKA) and the exchange protein directly activated by cAMP (Epac) signaling pathways (see parallel in this issue the in-depth review of CyaA-triggered signaling by Novak et al.). Briefly, in monocytes and macrophages, the CyaA-produced cAMP signaling through the cAMP/protein kinase A (PKA) pathway blocks the production of reactive oxygen species (ROS) and the complement-mediated uptake of opsonized particles already at very low (<10 ng/mL) CyaA concentrations [2,56,57]. At higher CyaA doses (100 ng/mL), FcR-mediated phagocytosis is also inhibited [57,58]. This appears to be primarily due to CyaA/cAMP-provoked near-instant inhibition of the activity of the non-receptor spleen tyrosine kinase (Syk) that is crucial in immune receptor signaling processes. Its inhibition by CyaA blocks signaling of the complement receptors required for opsonophagocytic uptake of bacteria [59] (Figure 2). Moreover, through cAMP/PKA-mediated inhibition of RhoA, the action of CyaA blocks macropinocytosis and triggers cell-exhausting but unproductive membrane ruffling [57]. 

In primary human neutrophils, a synergic activation of the PKA and Epac signaling pathways by the CyaA-produced cAMP blocks fMLF-activated oxidative burst and the production of ROS. Additionally, the activation of PKA triggers the inhibition of ERK1/2 and p38 MAP kinases. Activation of Epac by cAMP also blocks activation of the phospholipase C, with both the MAPKs and phospholipase C (PLC) activities being crucial for NADPH oxidase assembly and ROS production [6]. Activation of these pathways thus results in the blocking of opsonophagocytic uptake and killing of bacteria, the loss of chemotaxis and the lack of formation of bactericidal neutrophil extracellular traps (NETs) [2,60,61,62] (Figure 2). 

The enzymatic AC activity of CyaA also appears to extend the intracellular survival of non-opsonized internalized (invading) *B. pertussis* bacteria that enter into human and murine macrophages by a non-phagocytic mechanism [45,63,65]. CyaA-produced cAMP signaling in macrophages was shown to activate through the PKA pathway the Src homology domain 2 containing protein tyrosine phosphatase (SHP) 1, which plays a major role in regulation of receptor signaling in leukocytes. SHP-1 activation by CyaA then blocks expression of TLR-inducible NO synthase (iNOS) and thereby enables the internalized non-opsonized *B. pertussis* bacteria to evade NO-mediated intracellular killing [45,63] (Figure 2). 

Several studies have shown that CyaA can provoke macrophage apoptosis in vitro at toxin concentrations as low as 10 ng/mL [64,66,67]. Monocyte/macrophage apoptosis appears to be relevant in *B. pertussis* infection in vivo at least in the mouse model. CyaA produced by *B. pertussis* accounts for massive influx of phagocytes from the circulation into the infected mouse lung tissue, which provokes massive lung pathology and bacterial infiltration into lung parenchyma across the epithelial lining [67,68]. However, at the same time the toxin rapidly paralyses the bactericidal functions of incoming phagocytes and CyaA was shown to be the major virulence factor involved in host cell apoptosis in the infected mouse lungs [67]. The authors have observed apoptosis of macrophages and neutrophils both in BAL fluid and in the lung tissue after infection with wild-type *B. pertussis*, but not with a CyaA-deficient strain [67]. At physiologically relevant, nanomolar CyaA concentrations that appear to be close to those detected in nasopharyngeal washes from infected baboons and human infants [69,70], the pro-apoptotic signaling of CyaA is dependent on its adenylate cyclase activity. The macrophage apoptosis was shown to be rapidly triggered by the mitochondrial route, where cAMP-induced signaling of protein kinase A (PKA) and activation of tyrosine phosphatase SHP-1 leads to inhibition of MAPKs ERK1/2, thus yielding stabilization of BimEL and activation of Bax that triggers the mitochondrial apoptotic pathway [64] (Figure 2).

## 5. Effects of CyaA-Driven Intracellular cAMP Intoxication on DC Functions

**Figure 3:**
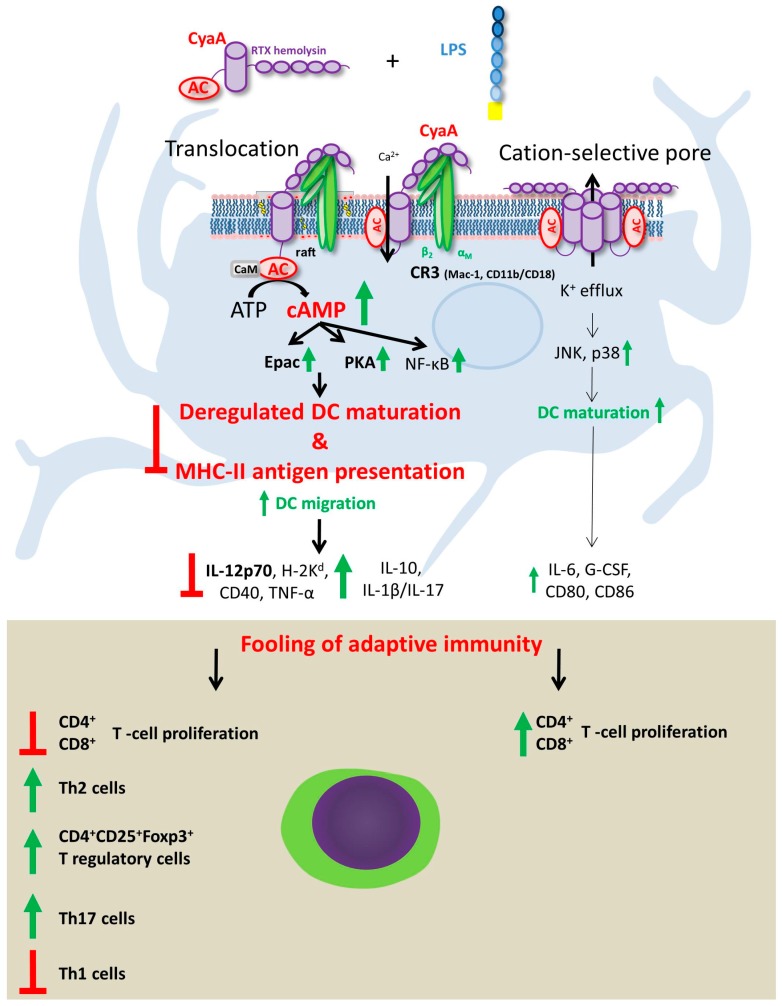
3′,5′-cyclic adenosine monophosphate (cAMP) signaling of CyaA subverts the functions of dendritic cells and fools the induction of T cell immune responses. CyaA/cAMP signaling in CR3-expressing (CD11b^+^) dendritic cells (DC) of the submucosa of the airways triggers an array of counteracting immunomodulatory signaling processes that fool induction of antigen-specific T cell immune responses by TLR-activated DC. cAMP signaling interferes with the proper maturation of DC and downregulates their capacity to process and present antigens to both CD4^+^ and CD8^+^ T cells. On the other hand, cAMP signaling enhances the migratory capacity of potentially tolerogenic DC that are impaired in IL-12 and TNFα secretion, while secreting enhanced amounts of IL-10 and exhibiting reduced expression of the MHC class II molecules and of the CD40 co-receptor. At least in vitro, CyaA-exposed DC can expand antigen-specific CD4^+^CD25^+^Foxp3^+^ T regulatory cells [71,72,73,74,75,76]. In parallel, concurrent cAMP signaling and permeabilization of DC by the CyaA pores and activation of NALP3 inflammasome due to potassium efflux-driven activation of JNK and p38 MAPK contributes secretion of IL-1β and of IL-17 [34]. As a result, the CyaA-hijacked DC are likely to contribute suppression of pro-inflammatory responses and delay activation of adaptive T- and B-cell mediated immunity to *B. pertussis* infection.

Given the pivotal role played by DC in all the different stages of the anti-infectious immune response, from pathogen recognition to T cells polarization, some of the most efficacious strategies of immune evasion employed by bacterial toxins evolved to subvert DC functions. This holds very true also for CyaA. Several studies have revealed how CyaA-driven intracellular cAMP intoxication may interfere with DC functions (Figure 3). 

A series of studies focused on intoxication of human monocyte derived DC (MDDC) or murine bone marrow derived DC (BMDC) with semi-purified CyaA. Bagley and coworkers found that CyaA induced the maturation of MDDC and that this effect correlated with the ability to induce intracellular cAMP [71]. However, those findings were likely biased by contamination of the used CyaA with lipopolysaccharide (LPS) and/or other TLR ligands. Another study utilized a more purified CyaA, which however exhibited quite low specific cell-penetrating activity. The CyaA-driven maturation of mouse BMDC was still found to be reduced upon addition of the LPS inhibitor polymyxin B, or when TLR4-deficient BMDC were used [72]. The authors hypothesized that a second signal through a TLR is required for the CyaA-induced activation of DC. Notably, both studies converged on the notion that action of CyaA on DC may inhibit their capacity to secrete IL-12 and TNF-α [71,72]. Since antigen-specific CD4^+^-T-cell clones generated from mice immunized with antigen and CyaA had cytokine profiles characteristic of Th2 or type 1 regulatory T (Tr1) cells, Ross and colleagues concluded that CyaA can promote Th2/Tr1-cell responses by inhibiting IL-12 and promoting IL-10 production [72]. 

**Figure 4:**
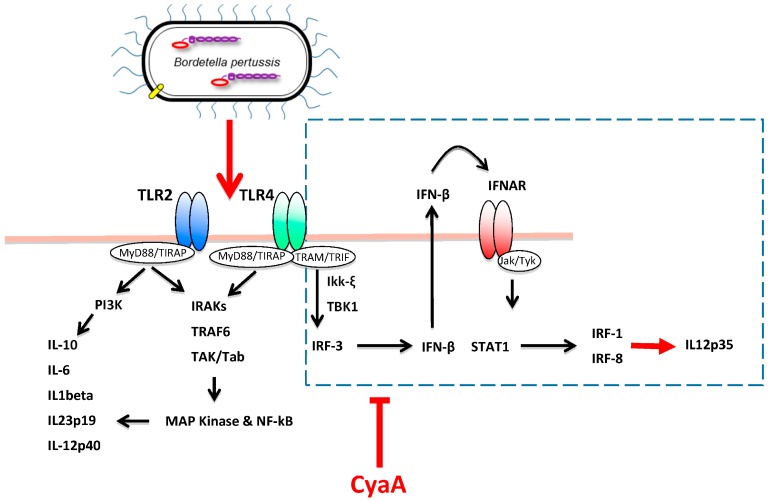
The CyaA toxin of B.pertussis inhibits the TRIF-dependent signaling pathways in human monocyte derived dendritic cells (MDDC). *B. pertussis* triggers TLR2 and TLR4 signaling in MDDC. In normal conditions, the MyD88-dependent signaling pathway downstream of TLR2 and TLR4 leads to the transcription of pro- and anti-inflammatory cytokines, including the p40 subunit of IL-12p70, while the TRIF-dependent signaling pathway downstream of TLR4 leads to the phosphorylation of interferon regulatory factor (IRF)3 and thereafter to the transcription of IFNβ and, through STAT1 phosphorylation, of IFN-inducible genes, including IRF1 and IRF8. A second wave of gene transcription is then activated, including the p35 subunit of IL-12p70 [77,78,79]. Upon encounter with *B. pertussis*, cellular intoxication with CyaA blocks the TRIF-dependent pathway in MDDC by inhibiting IRF3 phosphorylation and IFN-β, IRF-1, and IRF-8 expression [74,75].

The immunomodulatory activities that CyaA exerts on DC functions were next confirmed in infection experiments using CyaA-deficient *B. pertussis* strains. A first study showed that BMDC infected ex vivo with a CyaA-deficient *B. bronchiseptica* strain were able to produce increased levels of IL-12 as compared to the wild-type (wt) strain counterpart [73]. The authors showed that CyaA hampers IL-12 cytokine production through the inhibition of the p38 MAPK pathway. Ex vivo infection of MDDC with wt and CyaA-deficient *B. pertussis* helped to elucidate some of the mechanisms through which the toxin mediates immune evasion, in particular with regard to the modulation of IL-12 expression (Figure 4). 

Heterodimeric IL-12p70 represents a crucial physiological regulator of cellular immune response to microbial infections. Induction of the bioactive cytokine depends on the coordinated expression of the two genes composing IL-12p70, the p35 and the p40 subunits [77]. Studies conducted with an isogenic pair of wild type *B. pertussis* and its CyaA-deficient (*Bp18HS19*) mutant revealed that the adenylate cyclase toxin activity strongly interferes with human MDDC functions, blocking the production of heterodimeric IL-12p70 [74]. Indeed, infection of MDDC with wt *B. pertussis* induced partial maturation of DC but not secretion of IL-12. On the contrary, infection with the CyaA-deficient strain induced detectable levels of IL-12. In the same study, it was demonstrated that IL-12 secretion was dependent on the capacity of *Bp18HS19* to induce the expression of both the p40 and p35 subunits of IL-12, while the wt strain only induced the p40 subunit [74]. Blocking of p35 expression in *B. pertussis*-infected MDDC was shown to be mediated by CyaA-driven intracellular cAMP accumulation through the inhibition of interferon regulatory factor (IRF)-1 and IRF-8 expression [74]. These transcription factors are induced by type I IFN-α/β-mediated STAT-1 phosphorylation, which is activated by the TRIF-dependent pathway [78] and mediate IL-12p35 transcription [79] (Figure 4). 

It is worthy of note that the infection of MDDC with *Bp18HS19* induced, while the addition of exogenous cAMP to *Bp18HS19* infected MDDC inhibited, the expression of IFN-β, IRF-1 and IRF-8 [74]. As a result, MDDC infected with *Bp18HS19* drove a stronger Th1-oriented polarization of immune response as compared to MDDC infected by wt *B. pertussis* [74]. Notably, infection of MDDC with *Bp18HS19*, but not with wt *B. pertussis*, induced the phosphorylation of IRF-3, a master regulator of Type I IFN transcription [36]. The inhibition of the IRFs/IL-12p35 pathway by CyaA-induced cAMP was confirmed in the same study by the addition of enzymatically active CyaA toxin to *Bp18HS19* infected MDDC [36] and in murine BMDC treated with the purified toxin [75]. 

The MDDC infection model allowed addressing the role of the enzymatic activity of CyaA in the induction of a Th17 response. MDDC functions after encounter with wt *B. pertussis*, or with the *Bp18HS19* mutant, or with the CyaA-defective strain supplemented with either the fully functional highly purified CyaA, or a derivative lacking adenylate cyclase activity (CyaA-AC^−^) were compared. This approach allowed to demonstrate that CyaA expressed by *B. pertussis* strongly interferes with DC functions, reducing the expression of phenotypic markers and immunomodulatory cytokines and blocking IL-12p70 production. *B. pertussis*-treated MDDC promoted a mixed Th1/Th17 polarization, and the activity of CyaA altered the Th1/Th17 balance, enhancing Th17 and limiting Th1 expansion [36]. In the same study it was demonstrated that Th1 effectors are induced by *B. pertussis*-MDDC in the absence of IL-12p70 through an ERK1/2-dependent mechanism, and that p38 MAPK is essential for MDDC-driven Th17 expansion [36].

The role of CyaA toxin in shifting the immune system towards a Th17-type of response was further confirmed. Indeed, CyaA was shown to promote innate IL-1β production by BMDC via activation of the NALP3 inflammasome, which involves the cell-permeabilizing (pore-forming) activity of CyaA and polarizes the T cell response towards the Th17 subtype [34]. Interestingly, murine bone marrow-derived macrophages infected in vitro with a CyaA-deficient *B. bronchiseptica* strain induced lower levels of the Th17 driving cytokine IL-1β, and increased levels of the Th1 driving cytokine IL-12 p70, as compared to wild-type bacteria [80]. 

The effects of highly purified (TLR ligand-free) CyaA on murine and human DC, activated by addition of defined amounts of highly purified LPS as TLR4 ligand, were recently analyzed [80]. The results showed that signaling mediated by CyaA-induced cAMP has a major role in DC migration, since it enhanced TLR-induced dissolution of cell adhesive contacts and promoted migration of DC towards the lymph node-homing chemokines CCL19 and CCL21. When the capacity of toxin-treated DC to induce CD4^+^ and CD8^+^ T cell responses was analyzed, the exposure to CyaA was found to decrease the capacity of LPS-stimulated DC to present soluble protein antigen to CD4^+^ T cells independently of modulation of co-stimulatory molecules and of cytokine production. In parallel, the intoxication by cAMP enhanced the capacity of DC to promote expansion of CD4^+^CD25^+^Foxp3^+^ T regulatory cells in vitro [76]. In addition, CyaA decreased also the capacity of LPS-stimulated DC to induce antigen-specific CD8^+^ T cell proliferation and limited the induction of IFN-γ producing CD8^+^ T cells, while enhancing IL-10 and IL-17-production [76]. These results indicate that through activation of cAMP signaling, the CyaA toxin may be mobilizing intraepithelial DC that are impaired in T cell stimulatory capacity and the arrival of such potentially tolerogenic DCs into the draining lymph nodes might than contribute to a delay and to subversion of host immune responses in the course of *B. pertussis* infection.

## 6. Effects of Pore-Forming Activity of CyaA on DC Functions

The enzymatically inactive CyaA-AC^−^ toxoid, which still can permeabilize cells for potassium efflux in the absence of cAMP elevation, was shown to exhibit adjuvant activity and potentiate antibody production and T lymphocyte responses to co-administered *B. pertussis* antigens [81,82]. Moreover, co-administration of LPS-free CyaA-AC^−^ with a diluted commercial pertussis vaccine shifted the polarization of the resulting immune response from a Th2 profile to a mixed Th1/Th2 response [83]. This led to an improvement of mice protection against intranasal challenge by *B. pertussis,* well beyond the contribution of anti-CyaA antibodies [83]. These observations suggested that the CyaA-AC^−^ toxoid might exert its adjuvant activity through activation of DC by means of its intact pore-forming activity. Indeed, the pore-forming activity of CyaA-AC^−^ was shown to synergize with TLR signaling in promoting NALP3 inflammasome complex assembly and IL-1β secretion by DC [34]. The work of Dadaglio et al., 2014 showed that the adjuvant activity of high doses of endotoxin-free CyaA-AC^−^ toxoid on DC is mediated through TLR4/TRIF signaling by an as-yet unknown mechanism. Plausibly, the toxoid-induced relocation of the receptor CD11b/CD18 into lipid microdomains might yield its clustering with TLR4 and this might trigger TRIF signaling, even in the absence of endotoxin-mediated activation of TLR4 [84]. However, the CD8^+^ T response was not completely abolished in TLR4-/- and TRIF-/- mice in vivo, suggesting that the CyaA-AC^−^ toxoid may possess an additional adjuvant activity involved in stimulation of T cell responses by DC. This was shown to manifest already at low toxoid concentrations, where the pore-forming activity accounts for the intrinsic adjuvant activity of CyaA-AC^−^ DC in vitro and in vivo [85]. This adjuvant activity is independent of TLR2/4/9, TRIF, MyD88, CD14 and inflammasome signaling and depends on the capacity of the pores formed by CyaA-AC^−^ to elicit K^+^ efflux from cells. This activates p38 and JNK mitogen activated protein kinases (MAPKs), and the signaling via p38 and JNK MAPKs then induces the phenotypic maturation of DC and increases their capacity to migrate and stimulate antigen-specific responses of CD4^+^ and CD8^+^ T cells in vitro and in vivo [86] (Figure 3).

## 7. Future Perspective

Over the past 20 years, CyaA, and specifically the genetically detoxified CyaA-AC^−^ toxoid, have been exploited as non-replicative vectors for antigen delivery (reviewed recently in [9]). The toxoid has been shown to deliver antigenic T cell epitopes for processing both into the cytosolic as well the endosomal compartments of DC, enabling the subsequent loading of delivered epitopes onto MHC class I and class II molecules and their presentation to T cells on the surface of DCs. Recombinant CyaA-AC^−^ constructs could induce potent antigen-specific cytotoxic CD8^+^ T lymphocyte (CTL) responses against various antigens from viruses (e.g., HIV, CMV, LCMV, HPV or influenza), bacteria (*M. tuberculosis*), parasites (*P. berghei*), or tumors (melanoma or HPV-induced) [86,87]. Moreover, the CD11b-binding RTX hemolysin moiety of CyaA was shown to deliver into DC cytosol rather large antigens inserted within, or in place of, the AC domain [88,89,90,91]. 

Building on these studies, CyaA-derived toxoids carrying the E7 oncoantigen of human papilloma viruses 16 and 18 have now been proven to be safe in phase I and II clinical trials of immunotherapeutic CTL vaccines for treatment of papilloma virus-induced cervical cancer [92]. 

The CyaA-AC^−^ toxoid-based immunotherapeutics recently failed to meet the set endpoints in the phase II clinical trials of the cervical cancer vaccine (www.genticel.com). However, given the potent immunosuppressive action of the native CyaA toxin during *B. pertussis* infection and taking in account the capacity of CyaA to annihilate opsonophagocytic uptake and ROS-mediated killing of the bacteria, it appears of utmost importance to include CyaA toxoids into the future acellular pertussis vaccines. Induction of CyaA-neutralizing activities by the next generation of acellular pertussis vaccines appears to be the key to improvement of their performance beyond the current protection against severe disease caused by pertussis toxin in infants. The next generation of pertussis vaccines will also have to confer protection against infection of the vaccinated populations, to limit the circulation of the pathogen. Therefore, the toxoid of CyaA is currently considered a first choice antigen candidate for inclusion into the acellular pertussis vaccine.
